# High mortality of pneumonia in cirrhotic patients with ascites

**DOI:** 10.1186/1471-230X-13-25

**Published:** 2013-02-07

**Authors:** Tsung-Hsing Hung, Chih-Wei Tseng, Yu-Hsi Hsieh, Kuo-Chih Tseng, Chih-Chun Tsai, Chen-Chi Tsai

**Affiliations:** 1 Division of Gastroenterology, Department of Medicine, Buddhist Dalin Tzu Chi General Hospital, Chia-Yi, Taiwan; 2 School of Medicine, Tzu Chi University, Hualien, Taiwan; 3 Department of Mathematics, Tamkang University, Tamsui, Taiwan; 4 Division of Infectious disease, Department of Medicine, Buddhist Dalin Tzu Chi General Hospital, Chiayi, Taiwan

**Keywords:** Cirrhosis, Ascites, Infections, Pneumonia, Spontaneous bacterial peritonitis

## Abstract

**Background:**

Cirrhotic patients with ascites are prone to develop various infectious diseases. This study aimed to evaluate the occurrence and effect of major infectious diseases on the mortality of cirrhotic patients with ascites.

**Methods:**

We reviewed de-identified patient data from the National Health Insurance Database, derived from the Taiwan National Health Insurance Program, to enroll 4,576 cirrhotic patients with ascites, who were discharged from Taiwan hospitals between January 1, 2004 and June 30, 2004. We collected patients’ demographic and clinical data, and reviewed diagnostic codes to determine infectious diseases and comorbid disorders of their hospitalizations. Patients were divided into an infection group and non-infection group and hazard ratios (HR) were determined for specific infectious diseases.

**Results:**

Of the total 4,576 cirrhotic patients with ascites, 1,294 (28.2%) were diagnosed with infectious diseases during hospitalization. The major infectious diseases were spontaneous bacterial peritonitis (SBP) (645, 49.8%), urinary tract infection (151, 11.7%), and pneumonia (100, 7.7%). After adjusting for patients’ age, gender, and other comorbid disorders, the HRs of infectious diseases for 30-day and 90-day mortality of cirrhotic patients with ascites were 1.81 (1.54-2.11) and 1.60 (1.43-1.80) respectively, compared to those in the non-infection group. The adjusted HRs of pneumonia, urinary tract infection (UTI), spontaneous bacterial peritonitis (SBP), and sepsis without specific focus (SWSF) were 2.95 (2.05-4.25), 1.32 (0.86-2.05), 1.77 (1.45-2.17), and 2.19 (1.62-2.96) for 30-day mortality, and 2.57 (1.93-3.42), 1.36 (1.01-1.82), 1.51 (1.29-1.75), and 2.13 (1.70-2.66) for 90-day mortality, compared to those in the non-infection group.

**Conclusion:**

Infectious diseases increased 30-day and 90-day mortality of cirrhotic patients with ascites. Among all infectious diseases identified, pneumonia carried the highest risk for mortality.

## Background

Spontaneous bacterial peritonitis (SBP), pneumonia, and urinary tract infections (UTI) are the main infectious diseases found in cirrhotic patients [1-5]. According to a meta-analysis study, infections in cirrhotic patients increase mortality about four-fold [6]. SBP is the most thoroughly evaluated infection in cirrhotic patients. The prevalence of SBP among overall hospitalized patients with cirrhosis and ascites is estimated to 10% to 30% [7,8], and SBP accounts for at least 24% of overall bacterial infections [3-5,9,10]. In contrast, pneumonia was reported in about 21.4% of patients with end-stage liver disease with mortality rates as high as 37-41% [5,6]. In another study, community-acquired pneumonia was responsible for about 14.4% mortality in cirrhotic patients [11].

The above studies do not identify which infectious disease carried higher mortality in cirrhotic patients. In addition, cirrhotic patients develop SBP only when they have ascites. Since most cirrhotic patients enrolled in previous studies were unselected, the real effect of SBP and pneumonia on the mortality of cirrhotic patients still needs to be thoroughly evaluated. It would be more effective to compare the effect of different infectious diseases on the mortality of cirrhotic patients by evaluating only cirrhotic patients presenting with ascites. The purpose of this study was to determine the occurrence and influence of major infectious diseases on 30-day and 90-day overall mortality among cirrhotic patients with ascites.

## Methods

### Database

This study used patient data from the National Health Insurance Research Database in Taiwan, which is established and maintained by the Taiwan National Health Insurance Bureau and the National Health Research Institute. The Taiwan National Health Insurance program was developed in 1995; it includes all citizens residing in Taiwan and covers more than 98% of Taiwan’s population. The details of the database have been described in previous studies [12-14]. For the present study, the secondary de-identified database was used to identify all patient discharges in Taiwan. The study protocol was evaluated by the National Health Research Institute, and the privacy of the health care providers and patients were protected. This study was approved by the National Health Research Institute (application and agreement number: 100101).

### Study sample

This retrospective study included patients discharged from Taiwan hospitals with the diagnostic code of cirrhosis (ICD-9-CM code 571.5, or 571.2 in the database) between January 1, 2004 and June 30, 2004. Patients <30 years old were not included because the etiologies of cirrhosis for younger patients were much different from older adult patients. For patients who had more than one hospitalization in this period, only the first hospitalization was enrolled. Ascites was defined by ICD-9-CM code 789.5, or ICD-9 v3 Procedure Codes 54.91 in the database. We enrolled only cirrhotic patients with the diagnostic codes for ascites in their hospitalizations in the present study. For each patient enrolled, the starting point for evaluating the 30-day and 90-day mortality was the patients’ date of admission in enrolled hospitalization.

A total of 4,576 cirrhotic patients with ascites were identified and enrolled in this study. The patients were classified into an infection group and non-infection group according to the presence of infectious diseases or not in their enrolled hospitalizations. We reviewed all diagnostic codes of the enrolled patients to evaluate if they had infectious disease or not during their hospitalizations. The main infectious diseases in our study included pneumonia (ICD-9-CM codes 481–487, without 484), sepsis (ICD-9-CM codes 038, 020.0, 790.7, or 112.81) [15], urinary tract infection (UTI) (ICD-9-CM codes 590.1, 595.0, 595.9 or 599.0), and SBP. As in previous studies, SBP was defined as a patient with the ICD-9-CM diagnosis codes for both cirrhosis and peritonitis (codes 567.2, 567.8, or 567.9) [14,16,17]. In order to exclude secondary peritonitis, patients with other causes of peritonitis (such as appendicitis, ischemic bowel disease, biliary tract or hollow organ perforation), as well as those having an additional procedure code for abdominal surgery, were not included [14,16,17]. When patients had diagnostic codes of sepsis without other diagnostic codes for infectious focus, including catheter-related sepsis, they were considered to have sepsis without specific focus (SWSF). Other infectious diseases included cellulitis (ICD-9-CM code 681 or 682), biliary tract infection or acute cholecystitis (ICD-9-CM codes 576.1, 575.0, 574.00, 574.01, 574.30, 574.31, 574.60, 574.61, 574.80, 574.81), empyema (ICD-9-CM code 510), necrotizing fasciitis (ICD-9-CM code 728.86), septic arthritis (ICD-9-CM code 711), infective endocarditis (ICD-9-CM code 421), and other bacterial infections. A national mortality database was used to identify the dates of death and calculate patients’ overall 30-day and 90-day mortality.

### Statistical analyses

Cox hazard regressions were performed to evaluate the effect of infectious diseases on mortality in cirrhotic patients with ascites. Comorbid medical disorders selected for regression included age, gender, alcoholism (ICD-9-CM codes 291, 303, 305.00-305.03, 571.0-571.3), hepatocellular carcinoma (HCC) (ICD-9-CM code 155.0), hepatic encephalopathy (HE) (ICD-9-CM code 572.2), esophageal variceal bleeding (EBV) (ICD-9-CM codes 456.0, 456.20), renal function impairment (RFI) (ICD-9-CM codes 582, 585, 586, or 572.4), and peptic ulcer bleeding (PUB) (ICD-9-CM codes 531.0, 531.2, 531.4, 531.6, 532.0, 532.2, 532.4, 532.6, 533.0, 533.2, 533.4 and 533.6) [18]. Cox proportional hazard regressions were also performed to evaluate the effects of main infectious diseases on the mortality of cirrhotic patients with ascites compared to mortality in non-infection group. The Student’s t test was used to compare continuous variables, and the Chi square test was used to compare categorical variables. Hazard ratios (HR) and 95% confidence intervals (CI) using a significance level of 0.05 for this study were also calculated. The SPSS statistical package SPSS System for Windows, version 13.0 (SPSS, Chicago, IL, USA) was used to perform the analyses in this study.

## Results

Of the total 4,576 cirrhotic patients with ascites, mean age was 58.6 ± 13.7 years. There were 3,165 (69.2%) male patients and 1,411 (30.8%) female patients. During their enrolled hospitalizations, 1,294 patients had co-existence of infectious diseases and 3,282 did not. The clinical characteristics of both groups are shown in Table 1. More male patients were found in the non-infection group (66.2% vs. 70.4%, P = 0.006). Patients were aged 59.0 ± 13.8 years in the infection group and 58.4 ± 13.6 years in the non-infection group. No statistically significant differences were found between the both groups (P = 0.169). More patients with HCC were noted in the non-infection group than in the infection group (27.4% vs. 19.9%; P < 0.001). More patients had EVB in the non-infection group than in the infection group (14.5% vs. 10.2%; P < 0.001). More patients had alcohol-related cirrhosis in the non-infection group than in the infection group (20.6% vs. 17.9%; P = 0.039). Other factors, including HE, PUB, and RFI, were not different between the two groups.

**Table 1:** Comparison of demographic characteristics of the infection group (n = 1,294) and non-infection group (n = 3,282)

	**Infection (n = 1294)**	**Non-infection (n = 3282)**	***P *value**
Male, no. (%)	856 (66.2)	2309 (70.4)	0.006
Age, yr			0.169
30–44, no. (%)	219 (16.9)	572 (17.4)	
45–59, no. (%)	458 (35.4)	1154 (35.2)	
60–74, no. (%)	410 (31.7)	1100 (33.5)	
>75, no. (%)	207 (16.0)	456 (13.9)	
HCC, no. (%)	258 (19.9)	898 (27.4)	<0.001
Esophageal variceal bleeding	132 (10.2)	476 (14.5)	<0.001
Hepatic encephalopathy	218 (16.8)	488 (14.9)	0.095
PUB, no. (%)	64 (4.9)	192 (5.9)	0.231
Alcoholism, no. (%)	232 (17.9)	677 (20.6)	0.039
RFI, no. (%)	103 (8.0)	210 (6.4)	0.060

Abbreviations: HCC, hepatocellular carcinoma; PUB, peptic ulcer bleeding; RFI, renal function impairment.

Mortality in the two groups is shown in Table 2. In the non-infection group, the 30-day and 90-day mortality rates of cirrhotic patients with ascites were 12.7% and 26.0%, respectively. In the infection group, the 30-day and 90-day mortality rates of cirrhotic patients with ascites were 19.9% and 35.1%, respectively. The infection group included 645 (49.8%) patients with SBP, 188 (14.5%) patients with SWSF, 151 (11.7%) patients with UTI, and 100 (7.7%) patients with pneumonia. The remaining 210 (16.2%) patients had other infectious diseases or dual infections during their hospitalizations. The 30-day mortality rates of patients with SBP, SWSF, UTI, and pneumonia were 19.7%, 25.5%, 14.6%, and 32.0%, respectively. The 90-day mortality rates of patients with SBP, SWSF, UTI, and pneumonia were 33.5%, 44.7%, 31.8%, and 51.0%, respectively. Compared to SBP, pneumonia had higher 30-day (32.0% vs. 19.7%, P = 0.005) and 90-day (51.0% vs. 33.5%, P = 0.001) mortality.

**Table 2:** Comparison of 30-day and 90-day mortality of the infection group and non-infection group

**Infections**	**Number (%)**	**30-day mortality**	**90-day mortality**
No	3282 (71.7)	12.7%	26.0%
Yes	1294 (28.3)	19.9%	35.1%
SBP	645 (49.8)	19.7%	33.5%
UTI	151 (11.7)	14.6%	31.8%
Pneumonia	100 (7.7)	32.0%	51.0%
SWSF	188 (14.5)	25.5%	44.7%
Others	210 (16.2)	13.8%	26.2%

Abbreviations: SBP, spontaneous bacterial peritonitis; UTI, urinary tract infection; SWSF, sepsis without specific focus.

In order to alleviate the effects of other confounding factors, we used the Cox regression model to adjust the HRs of infectious diseases for the mortality of cirrhotic patients with ascites. Results are listed in Table 3. After adjusting for patients’ gender, age, and other medical comorbid disorders, the HRs of infectious diseases for 30-day and 90-day mortality were 1.81 (95% CI, 1.54-2.11; P < 0.001) and 1.60 (95% CI, 1.43-1.80; P < 0.001), respectively. Other risk factors for 30-day mortality of cirrhotic patients with ascites included age (HR, 1.02; 95% CI, 1.02-1.03, P < 0.001), male gender (HR, 1.49; 95% CI, 1.25-1.77; P < 0.001), RFI (HR, 2.70; 95% CI, 2.19-3.35; P < 0.001), EVB (HR, 1.52; 95% CI, 1.23-1.87; P < 0.001), HCC (HR, 2.30; 95% CI, 1.96-2.70; P < 0.001), and HE (HR, 1.28; 95% CI, 1.05-1.56; P = 0.013). Other risk factors for 90-day mortality of cirrhotic patients with ascites included age group (HR, 1.02; 95% CI, 1.02-1.03, P < 0.001), male gender (HR, 1.24; 95% CI, 1.09-1.40; P = 0.001), RFI (HR, 2.11; 95% CI, 1.78-2.51; P < 0.001), EVB (HR, 1.21; 95% CI, 1.03-1.43; P = 0.024), HCC (HR, 2.54; 95% CI, 2.26-2.84; P < 0.001), and HE (HR, 1.34; 95% CI, 1.16-1.54; P < 0.001). Alcohol-related cirrhosis correlated negatively with the 30-day and 90-day mortality of cirrhotic patients with ascites (HR, 0.77 and 0.78; 95% CI, 0.60-1.00 and 0.65-0.93; P, 0.045 and 0.007).

**Table 3:** Adjusted hazard ratios of risk factors for 30-day and 90-day mortality in cirrhotic patients with ascites

	**30-day mortality**		**90-day mortality**	
**Variable**	**HR (95% CI)**	***P *value**	**HR (95% CI)**	***P *value**
Infection	1.81 (1.54-2.11)	<0.001	1.60 (1.43-1.80)	<0.001
Age	1.02 (1.02-1.03)	<0.001	1.02 (1.02-1.03)	<0.001
Male	1.49 (1.25-1.77)	<0.001	1.24 (1.09-1.40)	0.001
RFI	2.70 (2.19-3.35)	<0.001	2.11 (1.78-2.51)	<0.001
Alcoholism	0.77 (0.60-1.00)	0.045	0.78 (0.65-0.93)	0.007
EVB	1.52 (1.23-1.87)	<0.001	1.21 (1.03-1.43)	0.024
HCC	2.30 (1.96-2.70)	<0.001	2.54 (2.26-2.84)	<0.001
PUB	1.16 (0.84-1.60)	0.374	1.13 (0.89-1.43)	0.322
HE	1.28 (1.05-1.56)	0.013	1.34 (1.16-1.54)	<0.001

Abbreviations: HR, hazard ratio; CI, confidence interval; RFI, renal function impairment; EVB, esophageal variceal bleeding; HCC, hepatocellular carcinoma; PUB, peptic ulcer bleeindg; HE, hepatic encephalopathy.

**Figure 1:**
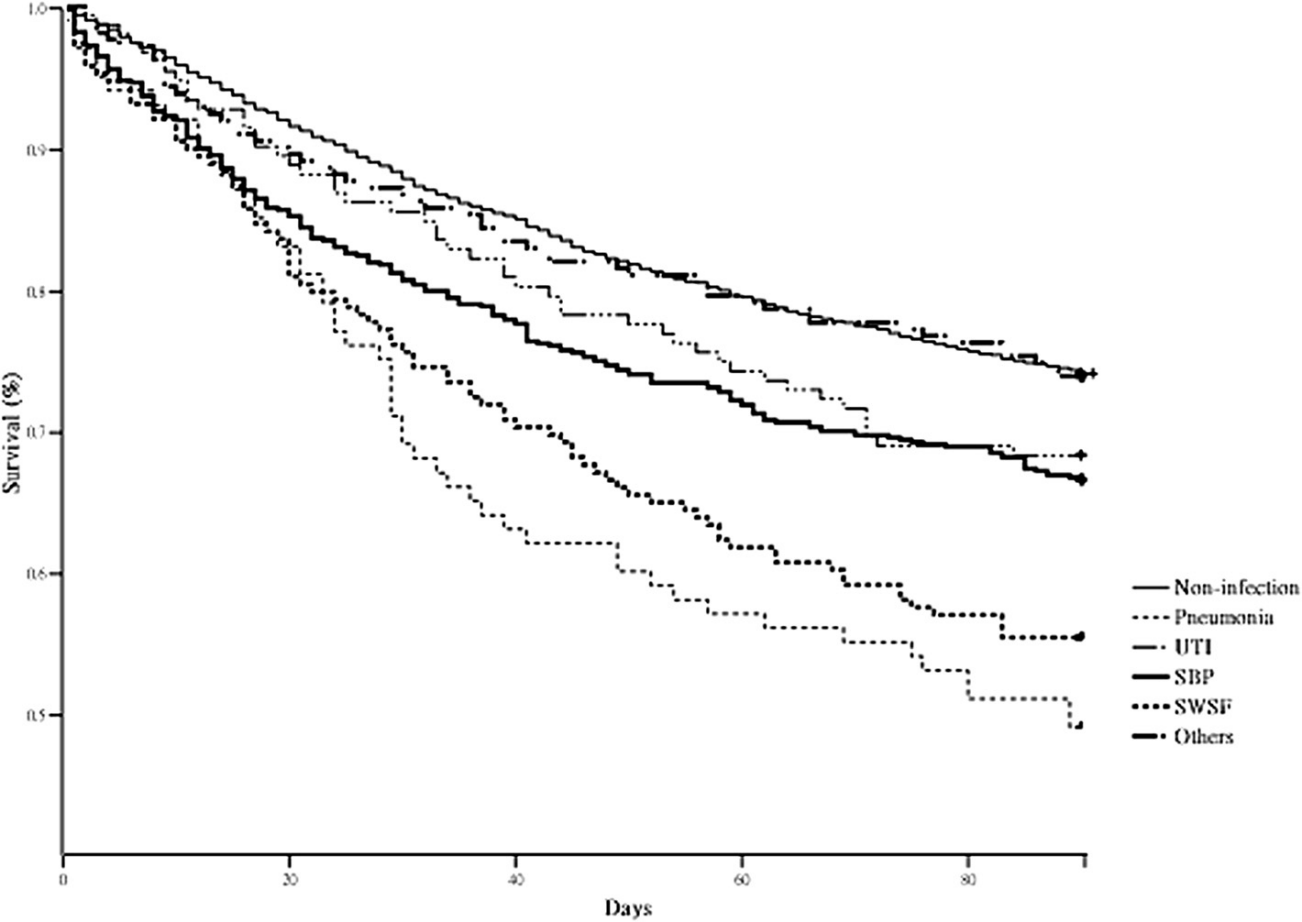
**Cumulative survival plot for cirrhotic patients with ascites with and without the presence of different infectious diseases.** After Cox regression model, the hazard ratios of pneumonia, urinary tract infection (UTI), spontaneous bacterial peritonitis (SBP), sepsis without specific focus (SWSF), and other infectious diseases for 90-day mortality were 2.57 (1.93-3.42), 1.36 (1.01-1.82), 1.51 (1.29-1.75), 2.13 (1.70-2.66), and 1.34(1.02-1.76) compared to non-infection group.

In order to compare the effect of different infectious diseases on the mortality of cirrhotic patients with ascites, we used the Cox regression model to adjust the HRs of different infectious diseases according to other confounding factors, including age group, gender, RFI, alcoholism, EVB, HCC, PUB, and HE. Results are shown in Table 4. Compared to the non-infection group, the adjusted HR of SBP, pneumonia, UTI, and SWSF was 1.77 (95% CI, 1.45-2.17; P < 0.001), 2.95 (95% CI, 2.02-4.25; P < 0.001), 1.32 (95% CI, 0.86-2.05; P = 0.207), and 2.19 (95% CI, 1.62-2.96; P < 0.001) for 30-day mortality. For 90-day mortality, the respective adjusted HRs were 1.51(95% CI, 1.29-1.75; P < 0.001), 2.57 (95%, 1.93-3.42; P < 0.001), 1.36 (95% CI, 1.01-1.82; P = 0.044), and 2.13 (95% CI, 1.70-2.66; P < 0.001). Pneumonia was responsible for the highest mortality risk among cirrhotic patients with ascites. Figure 1 shows the cumulative survival plot for cirrhotic patients with ascites with and without different infectious diseases.

**Table 4:** Adjusted hazard ratios (HR) of different types of infectious diseases for mortality of cirrhotic patients with ascites, compared with non-infection group

	**30-day mortality**		**90-day mortality**	
**Variables**	**HR (95% CI)**	***P *value**	**HR (95% CI)**	***P *value**
Pneumonia	2.95 (2.05-4.25)	<0.001	2.57 (1.93-3.42)	<0.001
UTI	1.32 (0.86-2.05)	0.207	1.36 (1.01-1.82)	0.044
SBP	1.77 (1.45-2.17)	<0.001	1.51 (1.29-1.75)	<0.001
SWSF	2.19 (1.62-2.96)	<0.001	2.13 (1.70-2.66)	<0.001
Others	1.56 (1.06-2.28)	0.023	1.34 (1.02-1.76)	0.039

Abbreviations: SBP, spontaneous bacterial peritonitis; UTI, urinary tract infection; SWSF, sepsis without specific focus.

## Discussion

Cirrhotic patients are prone to develop infectious diseases because of their underlying immune compromised status. In previous studies, SBP comprised about 20-30% of infectious diseases in cirrhotic patients [3-5]. However, most of these studies included a cirrhotic population without ascites. According to the results of the nationwide population-based database used in our study, about 28.3% of cirrhotic in-patients with ascites had infectious diseases, of which SBP comprised about 50%. Accordingly, when cirrhotic patients with ascites have septic signs, clinical physicians should consider SBP as a top candidate for infectious focus.

In the present study, pneumonia had the highest risk for mortality among cirrhotic patients with ascites, which was about 3-fold increased compared to the mortality of cirrhotic patients without infectious diseases. A previous study showed cirrhotic patients with pneumonia caused by *Staphylococcus aureus* had higher pneumonia severity index classes and demonstrated a higher mortality rate than non-cirrhotic patients [19]. Among pathogens for pneumonia in cirrhotic patients, the most frequent pathogen for community-acquired pneumonia is still *Streptococcus pneumonia*, and the predominant pathogens for hospital-acquired pneumonia are gram-negative bacilli and staphylococci [20]. This latter result is the same as that for pneumonia in non-cirrhotic patients, indicating that the pathogenesis of pneumonia in cirrhotic patients is not different from that in non-cirrhotic patients. Defects in innate pulmonary defenses caused by cirrhosis may explain why pneumonia contributes to the high mortality among cirrhotic patients [21,22]. Innate immunity is necessary for clearance of pathogens in patients with pneumonia even if they receive antibiotics [21-25]. High mortality of pneumonia in cirrhotic patients may be attributed to the poor ability of their innate immunity to clear the pathogens associated with pneumonia.

Because of the high mortality associated with pneumonia in cirrhotic patients, it is important for development of pneumonia to be avoided in these patients. Hepatic encephalopathy and tracheal intubations are usually considered to be risk factors for the development of pneumonia in cirrhotic patients. In addition, influenza infection can lead to hepatic decompensation, resulting in severe secondary bacterial pneumonia in cirrhotic patients [26,27]. In addition, plasma-based blood products increase transfusion-associated acute lung injury, which affects lung clearance and patients receiving these blood products are prone to develop pneumonia; red blood cell transfusions also increase the rate of pneumonia [28]. In order to prevent cirrhotic patients from developing pneumonia, it is critical to avoid needless transfusions and to prevent influenza infections.

In the present study, we found that SWSF also contributed to higher mortality among cirrhotic patients with ascites. We considered that this kind of infection was caused by a failure of patients’ defense mechanisms to prevent the microorganisms in the intestinal lumen from reaching the systemic circulation. When SWSF occurs, it means that the cirrhotic patients will have a tendency toward bacterial translocation from the intestinal lumen and poor clearance of bacteria in their bloodstream. This phenomenon may contribute to higher mortality among cirrhotic patients with ascites.

The very large sample size in the present study provided the statistical power to detect different effects of various infectious diseases on the mortality of cirrhotic patients with ascites. Nonetheless, some limitations of this study must be noted. First, although the severity of liver cirrhosis is commonly based by Child-Pugh scores or MELD scores, the database used in the present study did not allow us to identify the laboratory for bilirubin, albumin, creatinine, or prothrombin time by ICD-9 coding numbers. However, is has been proposed recently that the stages of cirrhosis are separated by easily defined clinical criteria [29]. The concept of four clinical stages of cirrhosis has been identified, and each stage has distinct clinical features and a markedly different prognosis. Cirrhotic patients with ascites are actually in decompensated status and are at least in stage 3 of the disease. On the other hand, complications of cirrhosis, such as EBV, HE, HCC, and RFI were considered in this study. Hence, we believe that lacking specific lab data is not a major flaw in this study, because all cases were reviewed in stage 3 according to the clinical staging system, and the other complications (such as EBV, HE, HCC, and RFI) were also well classified. Secondly, the exact etiology of liver cirrhosis was not identified in this national population-based study although nearly 20% of cirrhotic patients had alcohol-related cirrhosis. However, the etiology of liver cirrhosis in Taiwan has been well established in numerous published reports and is known to be related to the hepatitis B virus. However, the etiology of non-alcohol related liver cirrhosis cannot be confirmed definitively as a prognostic factor. Thirdly, the type of acquisition of infection is important in understanding the mortality of infection. Unfortunately, we were not able to identify exactly if the infections were community-acquired or hospital-acquired. Fourthly, systemic inflammatory response syndrome (SIRS) is considered to be a negative prognostic factor in patients with advanced cirrhosis [30]. Due to the limitations of our database, we could not identify if the cirrhotic patients had SIRS during their hospitalizations. Finally, even using this database, we could not identify sufficient numbers of other rare infectious diseases for comparison, including necrotizing fasciitis, meningitis, infective endocarditis, etc. However, these infections may have less clinical significance because of the limited number of cases encountered in clinical practice. Despite these limitations, this study is the first complete nationwide population-based study for identifying the occurrence and mortality risk of major infections in cirrhotic patients with ascites.

## Conclusions

In summary, the presence of infectious diseases increases 30-day mortality about 1.8-fold in cirrhotic patients with ascites. SBP is responsible for nearly half of infections in cirrhotic patients with ascites. Pneumonia carries the highest risk for overall 30-day and 90-day mortality among cirrhotic patients with ascites.

## Competing interests

The authors declare that they have no competing interests.

## Authors’ contributions

TH and CC* participated in design of the study, and performed the statistical analysis, and prepared the manuscript. CW, YH, and KC were consultants for medical information. CC was a consultant for statistical analyses. All authors read and approved the final manuscript.

## Pre-publication history

The pre-publication history for this paper can be accessed here:

http://www.biomedcentral.com/1471-230X/13/25/prepub

## References

[B1] Christou L Pappas G Falagas ME Bacterial infection-related morbidity and mortality in cirrhosis Am J Gastroenterol 2007 102 1510 1517 10.1111/j.1572-0241.2007.01286.x 17509025

[B2] Bonnel AR Bunchorntavakul C Reddy KR Immune dysfunction and infections in patients with cirrhosis Clin Gastroenterol Hepatol 2011 9 727 738 10.1016/j.cgh.2011.02.031 21397731

[B3] Fernández J Navasa M Gómez J Colmenero J Vila J Arroyo V Rodés J Bacterial infections in cirrhosis: epidemiological changes with invasive procedures and norfloxacin prophylaxis Hepatology 2002 35 140 148 10.1053/jhep.2002.30082 11786970

[B4] Borzio M Salerno F Piantoni L Cazzaniga M Angeli P Bissoli F Boccia S Colloredo-Mels G Corigliano P Fornaciari G Marenco G Pistarà R Salvagnini M Sangiovanni A Bacterial infection in patients with advanced cirrhosis: a multicentre prospective study Dig Liver Dis 2001 33 41 48 10.1016/S1590-8658(01)80134-1 11303974

[B5] Caly WR Strauss E A prospective study of bacterial infections in patients with cirrhosis J Hepatol 1993 18 353 358 10.1016/S0168-8278(05)80280-6 8228129

[B6] Arvaniti V D’Amico G Fede G Manousou P Tsochatzis E Plequezuelo M Burroughs AK Infections in patients with cirrhosis increase mortality four-fold and should be used in determining prognosis Gastroenterology 2010 139 1246 1256 10.1053/j.gastro.2010.06.019 20558165

[B7] Runyon BA Low-protein-concentration ascitic fluid is predisposed to spontaneous bacterial peritonitis Gastroenterology 1986 91 1343 1346 3770358 10.1016/0016-5085(86)90185-x

[B8] Pinzello G Simonetti RG Craxi A Di Piazza S Spanò C Pagliaro L Spontaneous bacterial peritonitis: a prospective investigation in predominantly nonalcoholic cirrhotic patients Hepatology 1983 3 545 549 6862365 10.1002/hep.1840030411

[B9] Tandon P Garcia-Tsao G Bacterial infections, sepsis, and multiorgan failure in cirrhosis Semin Liver Dis 2008 28 26 42 10.1055/s-2008-1040319 18293275

[B10] Tandon P Garcia-Tsao G Renal dysfunction is the most important independent predictor of mortality in cirrhotic patients with spontaneous bacterial peritonitis Clin Gastroenterol Hepatol 2011 9 260 265 10.1016/j.cgh.2010.11.038 21145427 PMC3713475

[B11] Viasus D Garcia-Vidal C Castellote J Adamuz J Verdaquer R Dorca J Manresa F Gudio F Carratalà J Community-acquired pneumonia in patients with liver cirrhosis: clinical features, outcomes, and usefulness of severity scores Medicine 2011 90 110 118 10.1097/MD.0b013e318210504c 21358441

[B12] Wu CY Kuo KN Wu MS Chen YJ Wang CB Lin JT Early Helicobacter pylori eradication decreases risk of gastric cancer in patients with peptic ulcer disease Gastroenterology 2009 137 1641 1648 10.1053/j.gastro.2009.07.060 19664631

[B13] Hung TH Hsieh YH Tsai CC Tseng KC Tsai CC Is liver cirrhosis a risk factor for osteonecrosis of the femoral head in adults? A population-based 3-year follow-up study Intern Med 2011 50 2563 2568 10.2169/internalmedicine.50.5952 22041357

[B14] Hung TH Tsai CC Hsieh YH Tsai CC Tseng CW Tsai JJ Effect of renal impairment on mortality of patients with cirrhosis and spontaneous bacterial peritonitis Clin Gastroenterol Hepatol 2012 10 677 681 10.1016/j.cgh.2012.02.026 22391345

[B15] Martin GS Mannino DM Eaton S Moss M The epidemiology of sepsis in the United States from 1979 through 2000 N Engl J Med 2003 348 1546 1554 10.1056/NEJMoa022139 12700374

[B16] Thuluvath PJ Morss S Thompson R Spontaneous bacterial peritonitis–in-hospital mortality, predictors of survival, and health care costs from 1988 to 1998 Am J Gastroenterol 2001 96 1232 1236 11316175 10.1111/j.1572-0241.2001.03708.x

[B17] Ko CW Kelley K Meyer KE Physician specialty and the outcomes and cost of admissions for end-stage liver disease Am J Gastroenterol 2001 96 3411 3418 10.1111/j.1572-0241.2001.05343.x 11774958

[B18] Wu CY Wu MS Kuo KN Wang CB Chen YJ Lin JT Long-term peptic ulcer rebleeding risk estimation in patients undergoing haemodialysis: a 10-year nationwide cohort study Gut 2011 60 1038 1042 10.1136/gut.2010.224329 21266725

[B19] Kang CI Song JH Ko KS Chung DR Peck KR Asian Network for Surveillance of Resistant Pathogens (ANSORP) Study Group Clinical significance of Staphylococcus aureus infection in patients with chronic liver diseases Liver Int 2010 30 1333 1338 10.1111/j.1478-3231.2010.02270.x 20492505

[B20] Cheruvattath R Balan V Infections in patients with end-stage liver disease J Clin Gastroenterol 2007 41 403 411 10.1097/01.mcg.0000248018.08515.f9 17413611

[B21] Propst-Graham KL Preheim LC Vander Top EA Snitily MU Gentry-Nielsen MJ Cirrhosis-induced defects in innate pulmonary defenses against Streptococcus pneumoniae BMC Microbiol 2007 7 94 10.1186/1471-2180-7-94 17956621 PMC2140065

[B22] Ono Y Watanabe T Matsumoto K Ito T Kunii O Goldstein E Opsonophagocytic dysfunction in patients with liver cirrhosis and low responses to tumor necrosis factor-alpha and lipopolysaccharide in patients’ blood J Infect Chemother 2004 10 200 207 15365859 10.1007/s10156-004-0321-7

[B23] Eddens T Kolls JK Host defenses against bacterial lower respiratory tract infection Curr Opin Immunol 2012 24 424 430 10.1016/j.coi.2012.07.005 22841348 PMC3971433

[B24] Gentry MJ Snitily MU Preheim LC Phagocytosis of Streptococcus pneumonia measured in vitro and in vivo in a rat model of carbon tetrachloride-induced liver cirrhosis J Infect Dis 1995 171 350 355 10.1093/infdis/171.2.350 7844371

[B25] Mueller-Ortiz SL Drouin SM Wetsel RA The alternative activation pathway and complement component C3 are critical for a protective immune response against Pseudomonas aeruginosa in a murine model of pneumonia Infect Immun 2004 72 2899 2906 10.1128/IAI.72.5.2899-2906.2004 15102802 PMC387850

[B26] Duchini A Viernes ME Nyberg LM Hendry RM Pockros PJ Hepatic decompensation in patients with cirrhosis during infection with influenza A Arch Intern Med 2000 160 113 115 10.1001/archinte.160.1.113 10632312

[B27] Marzano A Marengo A Ruggiero T Allice T Sanna C Alessandria C Morgando A Sciandrello MC Franzin AM Rizzetto M Ghisetti V Clinical impact of A/H1/N1/09 influenza in patients with cirrhosis: experience from a nosocomial cluster of infection J Med Virol 2013 85 1 7 10.1002/jmv.23454 23154873

[B28] Benson AB Burton JR Jr Austin GL Biggins SW Zimmerman MA Kam I Mandell S Silliman CC Rosen H Moss M Differential effects of plasma and red blood cell transfusions on acute lung injury and infection risk following liver transplantation Liver Transpl 2011 17 149 158 10.1002/lt.22212 21280188 PMC3399914

[B29] D’Amico G Garcia-Tsao G Pagliaro L Natural history and prognostic indicators of survival in cirrhosis: a systematic review of 118 studies J Hepatol 2006 44 217 231 10.1016/j.jhep.2005.10.013 16298014

[B30] Abdel-Khalek EE El-Fakhry A Helaly M Hamed M Elbaz O Systemic inflammatory response syndrome in patients with liver cirrhosis Arab J Gastroenterol 2011 12 173 177 10.1016/j.ajg.2011.11.006 22305496

